# Neuroprotective Potential of *Hericium erinaceus* Through Modulation of Inflammatory Signaling in THP-1 Macrophages Under Low-Level Lead Exposure

**DOI:** 10.3390/ijms27031318

**Published:** 2026-01-28

**Authors:** Patrycja Kupnicka, Izabela Szućko-Kociuba, Alicja Trzeciak-Ryczek, Michalina Ptak, Katarzyna Piotrowska, Maciej Kołodziejczak, Irena Baranowska-Bosiacka

**Affiliations:** 1 Department of Biochemistry and Medical Chemistry, Pomeranian Medical University in Szczecin, Al. Powstańców Wlkp. 72, 70-111 Szczecin, Poland; 2 Institute of Biology, University of Szczecin, 13 Wąska, 71-415 Szczecin, Poland; 3 The Centre for Molecular Biology and Biotechnology, University of Szczecin, 13 Wąska, 71-415 Szczecin, Poland; 4 Department of Physiology, Pomeranian Medical University in Szczecin, Al. Powstańców Wlkp. 72, 70-111 Szczecin, Poland

**Keywords:** *Hericium erinaceus*, inflammation, macrophage, neurodegeneration, neuroprotection, immunomodulation

## Abstract

Exposure to lead is associated with microglial dysfunction and the development of neuroinflammation. This contributes to accelerated neurodegeneration. Even low doses of this element modulate inflammatory responses and might contribute to central nervous system dysfunction. Extracts from the mushroom *Hericium erinaceus* (HE) possess well-documented neurotropic properties; however, its potential neuroprotective mechanisms under conditions of environmental neurotoxicity remain poorly defined. In this study, we investigated the effects of HE on inflammatory signaling in a microglia-oriented in vitro model using THP-1-derived macrophages exposed to low levels of lead (3.5 µg/dL). In our study, Pb exposure did not increase tumor necrosis factor (TNF) alpha levels but reduced monocyte chemoattractant protein-1 (MCP-1) secretion and altered cyclooxygenase (COX) expression, indicating immune response modulation rather than inflammatory activation. Under combined Pb and HE exposure, a marked shift in cyclooxygenase expression toward COX-2 at both the gene and protein levels was observed, accompanied by increased PGE_2_ production; these effects were dose-dependent. The inflammatory signaling was modulated rather than amplified. Also, TNF alpha levels were elevated after combined treatment, whereas gene expression responses were dose-dependent. MCP-1 secretion was fine-tuned toward control values, consistent with macrophage morphological changes, while IL-6 levels were increased. Overall, these findings indicate that *Hericium erinaceus* exerts immunomodulatory effects in microglia-like cells under low-level lead exposure, supporting its neuroprotective potential through modulation of neuroinflammatory signaling.

## 1. Introduction

Neuroinflammation is a response of the central nervous system (CNS) to disturbances in homeostasis. This process is primarily mediated by glial cells, with microglia playing a central role. As the resident innate immune cells of the CNS, functioning as resident macrophages, microglia are highly sensitive to even minor pathological changes. Through the release of cytokines and growth factors, microglia can initiate both neurotoxic and reparative responses [1,2].

Neuroinflammation may be elicited by diverse factors, including infection and trauma, but it can also result from exposure to environmental agents such as heavy metals [3]. Among these, lead (Pb) is a major contributor to neuroinflammatory processes. This element is classified as an environmental pollutant due to its elevated concentrations in air, soil, water, and food [4,5]. Additionally, industrial activities release Pb nanoparticles into the atmosphere, posing a substantial risk to human health [6]. The Centers for Disease Control and Prevention (CDC) has established a blood Pb reference value of 3.5 µg/dL; however, the agency stresses that no level of Pb in the blood can be considered safe [7]. Adverse effects occur even at relatively low blood Pb concentrations, particularly in vulnerable populations such as children [8,9]. Pb exposure is associated with neurodysfunction and contributes to the development of neurodegenerative diseases [10], including Alzheimer’s disease [11] and Parkinson’s disease [12].

Exposure to Pb, especially at higher levels, has been shown to increase the expression of proinflammatory mediators and signaling molecules in multiple brain regions. These include interleukins (IL-1β, IL-6, IL-18), TNF-alpha, nuclear factor kappa B (NF-κB), cyclooxygenases (COX), and transforming growth factor β (TGFβ), produced by both microglia and astrocytes [13]. In parallel, Pb exposure promotes oxidative stress in microglia, resulting in increased generation of reactive oxygen species (ROS), enhanced cytokine production, and activation of nuclear factor erythroid 2-related factor 2 (Nrf2) signaling pathways [14,15].

Cytokines released during neuroinflammation play a critical role in regulating inflammatory responses, intracellular signaling, and neuronal growth and survival, thereby linking neuroinflammatory activity with neurodegenerative processes. Under physiological conditions, cytokine secretion in the healthy brain creates a tightly controlled, localized inflammatory environment that supports microglial recruitment and facilitates efficient clearance of pathogens or tissue damage [16]. In addition to cytokines, chemokines such as monocyte chemoattractant protein-1 (MCP-1) are essential regulators of microglial recruitment, migration, and immune surveillance within the CNS [17].

Exposure to Pb has also been shown to impair the phagocytic capacity of macrophages [18], while low concentrations of this metal may exert immunosuppressive effects [19,20]. Such Pb-induced immune dysregulation can compromise microglial surveillance, weakening endogenous neuroprotective mechanisms. Concurrently, persistent inflammation favors the accumulation of misfolded and aggregated proteins in neural cells, further amplifying neuroinflammatory signaling and accelerating neurodegenerative pathology [16]. Proper microglial function is therefore essential for maintaining neuronal homeostasis and for the effective clearance of β-amyloid plaques, highlighting the need for a finely regulated balance between protective and detrimental neuroinflammatory responses [16]. Notably, certain cytokines, including IL-6, exhibit pleiotropic effects in the CNS and may contribute to glial communication and tissue repair, depending on the cellular and molecular context [21].

Within the inflammatory process, cyclooxygenases are key enzymes that catalyze the conversion of arachidonic acid into local mediators, including prostaglandin E_2_ (PGE_2_) and thromboxane A_2_ (TXA_2_), which is transformed into thromboxane B_2_ (TXB_2_) [22]. Under physiological conditions, the constitutive activity of cyclooxygenase-1 (COX-1) predominates [23]. Cyclooxygenase-2 (COX-2) is an inducible enzyme, whose expression increases dramatically in response to inflammatory stimuli [24,25]. A wide range of stimuli to which glial cells respond can differentially regulate cyclooxygenase activity, thereby shaping and diversifying the inflammatory response [26].

Another factor, TNF alpha, is a potent inducer of COX-2 expression through the activation of NF-κB and mitogen-activated protein kinase (MAPK) signaling pathways, leading to enhanced PGE_2_ synthesis and amplification of the local inflammatory response [27]. This mechanism may also act in the opposite direction, whereby cyclooxygenase-derived products promote the upregulation of TNF alpha expression [28,29]. However, prostaglandins might also contribute to the decrease in the mRNA expression of *TNF alpha* [30].

Modulators of the inflammatory response can influence the progression of neuroinflammation and the associated neurodegeneration. One factor with notable neuroprotective potential is *Hericium erinaceus* (HE), a medicinal fungus recognized for its potent bioactive properties. Its extracts contain erinacines, hericenones, polysaccharides, and dilinoleyl-phosphatidylethanolamine, which exhibit multiple therapeutic effects, including antioxidative [31], immunomodulatory, and anti-inflammatory activities [32]. Moreover, *H. erinaceus* has been shown to stimulate the synthesis of nerve growth factor (NGF), inhibit β-amyloid (Aβ) cytotoxicity, and protect neurons against oxidative stress-induced cell death [33]. Beneficial effects of HE have been reported in the management of cognitive impairment, Alzheimer’s disease, ischemic stroke, Parkinson’s disease, and age-related hearing loss [33].

Considering the anti-inflammatory and immunomodulatory properties of HE extracts, they have been proven to inhibit TNF alpha-induced angiogenesis and ROS generation via modulation of matrix metalloproteinase-9 (MMP-9)/NF-κB and Nrf2 signaling in human EA.hy926 endothelial cells, as well as contribute to the reduction in COX-2 expression in C57BL mice astrocytes [34,35].

To date, no published studies have examined the impact of HE extracts on cyclooxygenase expression in macrophages or microglial cells. In this study, we explored the neuroprotective potential of HE extracts in THP-1–derived macrophages exposed to a low concentration of Pb (3.5 µg/dL). The selected in vitro model has previously been employed as a microglia-like system in studies investigating the mechanisms of neuroinflammation [36,37,38]. In our research, we aim to link these neuromodulatory properties with the potential neuroprotective activity of *H. erinaceus*.

## 2. Results

### 2.1. Hericium erinaceus Cytotoxicity in THP-1 Macrophages

**Figure 1:**
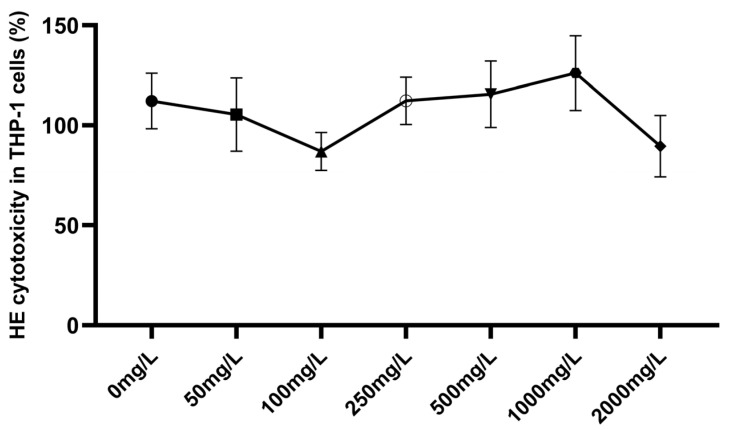
HE extract in THP-1 macrophages (% viability). THP-1 macrophages were cultured in complete medium supplemented with HE extract at concentrations of 0, 50, 100, 250, 500, 1000, or 2000 mg/L. Cytotoxicity was assessed using the WST-1 reagent. One-way ANOVA followed by Dunnett’s post hoc test was applied. No statistically significant differences were observed compared with the 0 mg/L control group.

In the cytotoxicity assessment of HE extract at concentrations ranging from 0 to 2000 mg/L in THP-1 macrophages, no statistically significant changes in cell viability were observed. At concentrations of 50–100 mg/L, cell viability exhibited a decreasing trend, with mean reductions of 6% and 22.5%, respectively. In contrast, at concentrations of 250–1000 mg/L, viability showed an increasing trend; however, these differences were not statistically significant (Figure 1).

### 2.2. Morphology of THP-1 Macrophages

**Figure 2:**
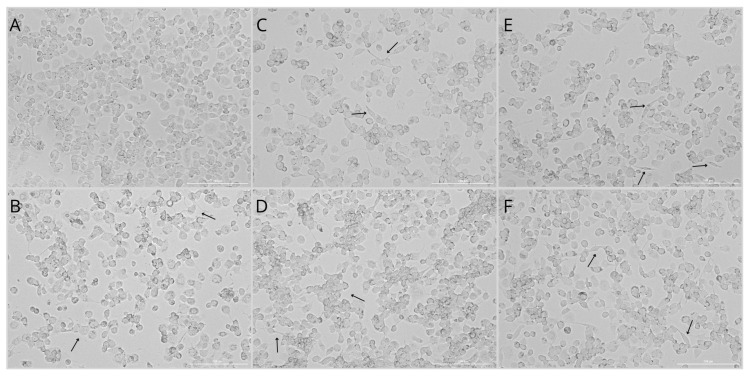
Representative bright-field images of THP-1–derived macrophages from the control group (**A**) and experimental groups: exposed to 3.5 µg/dL Pb (**B**), treated with 250 mg/L (**C**) or 500 mg/L (**D**) HE extract, and exposed to 3.5 µg/dL Pb combined with 250 mg/L (**E**) or 500 mg/L (**F**) HE extract. Arrows indicate characteristic morphological features of the cells, including differences in shape, granularity, and spatial organization, corresponding to phenotypic variation between M1-like (flattened, irregular, granular) and M2-like (elongated, spindle-shaped, with smooth contours) macrophages. Scale bar = 200 µm.

The control group (Figure 2A) exhibited marked heterogeneity and a disorganized spatial distribution of cells within the culture. Cells displayed short protrusions, a flattened appearance, and irregular shapes. In contrast, cultures exposed to Pb (Figure 2B) showed increased cytoplasmic granularity; they were predominantly round to oval and exhibited numerous projections.

Macrophages treated with HE extract (Figure 2C,D) demonstrated reduced cytoplasmic granularity and a more elongated, spindle-like morphology. Notably, cells treated with 500 mg/L of the extract formed compact and more regularly arranged clusters. In the Pb + 250HE group, cells displayed enhanced cytoplasmic granularity, although this effect was less pronounced than in the Pb-only group (Figure 2E). This culture was characterized by a higher proportion of elongated cells. Macrophages in the Pb + 500HE group also exhibited cytoplasmic granules; however, a greater number of elongated, spindle-shaped cells was observed, and the overall cellular organization appeared more regular (Figure 2F). The morphology of this group was comparable to that observed in cultures treated with HE extract alone (Figure 2C,D).

These morphological differences may suggest a shift from a pro-inflammatory phenotype, characterized by irregular and granular cells, toward a less inflammatory phenotype, marked by elongated cells with reduced cytoplasmic granularity, particularly in cultures treated with HE extract.

### 2.3. Cytokines and Chemokines

#### 2.3.1. *TNF alpha* mRNA Expression

**Figure 3:**
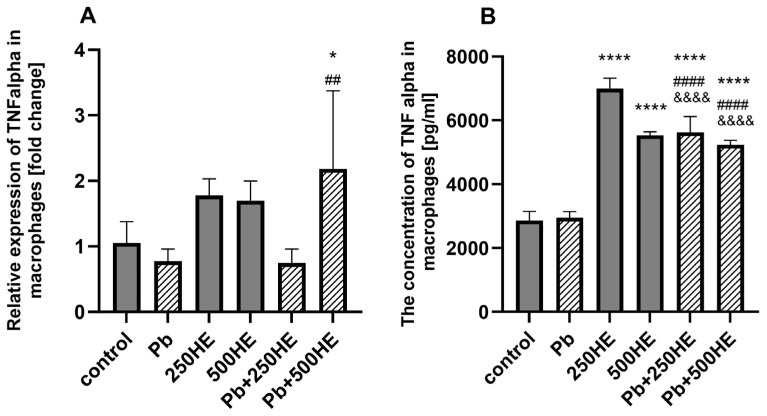
Relative mRNA expression of *TNF alpha* in macrophages (**A**) and TNF alpha concentration in macrophage culture medium (**B**) in the control group and experimental groups: exposed to 3.5 µg/dL Pb, treated with 250 mg/L or 500 mg/L *Hericium erinaceus* extract (250HE and 500HE), and exposed to 3.5 µg/dL Pb combined with 250 mg/L or 500 mg/L *Hericium erinaceus* extract. Each group included six samples. Data are presented as mean ± SD. One-way ANOVA followed by Tukey’s multiple comparisons post hoc test: * *p* < 0.05, **** *p* < 0.0001 vs. control; ## *p* < 0.005; #### *p* < 0.0001 vs. Pb; &&&& *p* < 0.0001 vs. corresponding HE group.

The expression of the *TNF alpha* gene in macrophages was upregulated by the addition of 250 mg/L and 500 mg/L HE extract vs. control; however, the difference was not significant. When incubated with 3.5 µg/dL of Pb, the expression of *TNF alpha* decreased; however, the difference was not significant. When combined with 250 mg/L of HE, the mRNA expression of *TNF alpha* gene was lower than in other groups (non-statistical); however, the addition of 500 mg/L HE reversed potential Pb inhibition of this gene and upregulated the expression by 107% percent vs. control (*p* = 0.0160) and 182% vs. Pb (*p* = 0.0018) (Figure 3A).

#### 2.3.2. TNF Alpha Concentration in Culture Media

The concentration of TNF alpha in the macrophage culture medium was not affected by the addition of 3.5 µg/dL of Pb. In contrast, incubation of the cells with HE extract led to an increased production of this cytokine. The 250HE group showed an 144% increase (*p* < 0.0001), while the 500HE group exhibited an 94% increase (*p* < 0.0001). Elevated TNF alpha levels were also observed in groups exposed to Pb and treated with HE extracts. Both 250 mg/L and 500 mg/L of the extract significantly increased TNF alpha levels (*p* < 0.0001, for both); however, the increase was lower compared to the corresponding groups not exposed to Pb (*p* < 0.0001) (Figure 3B).

#### 2.3.3. MCP-1 Concentration in Macrophage Culture Media

**Figure 4:**
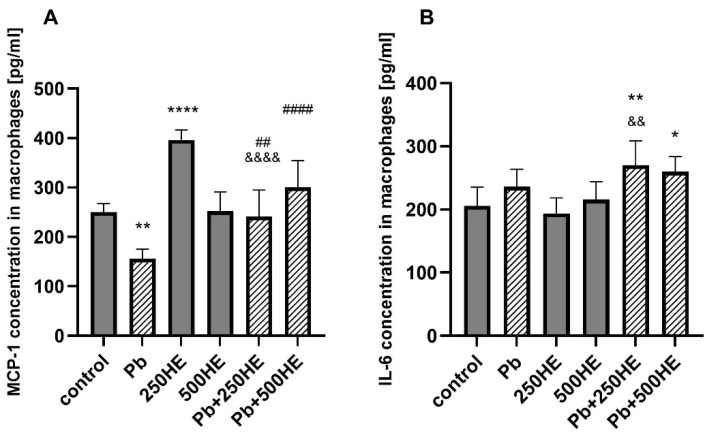
The concentration of MCP-1 (**A**) and IL-6 (**B**) in macrophage culture medium of the control group and experimental groups: exposed to 3.5 µg/dL Pb, treated with 250 mg/L or 500 mg/L *Hericium erinaceus* extract (250HE and 500HE), and exposed to 3.5 µg/dL Pb combined with 250 mg/L or 500 mg/L *Hericium erinaceus* extract. Each group included six samples. Data are presented as mean ± SD. One-way ANOVA followed by Tukey’s multiple comparisons post hoc test: * *p* < 0.05, ** *p* < 0.005, **** *p* < 0.0001 vs. control; ## *p* < 0.005; #### *p* < 0.0001 vs. Pb; && *p* < 0.005; &&&& *p* < 0.0001 vs. corresponding HE group.

Pb exposure resulted in a decrease in MCP-1 concentration compared with the control group (*p* = 0.0017). Treatment with HE extracts alone showed dose-dependent effects: 250 mg/L HE significantly increased MCP-1 levels relative to control (*p* < 0.0001), whereas 500 mg/L HE did not induce a significant change. In Pb-exposed macrophages, co-treatment with both 250 mg/L and 500 mg/L HE restored Pb-suppressed MCP-1 secretion, with the higher extract concentration showing a stronger effect (Figure 4A).

#### 2.3.4. Il-6 Concentration in Macrophage Culture Media

Pb exposure alone did not significantly affect IL-6 secretion compared with control macrophages, similar to the treatment with HE extracts. In Pb-exposed cells, co-treatment with HE extract led to an increase in IL-6 secretion, reaching statistical significance for both Pb + 250HE and Pb + 500HE groups (*p* = 0.0071, *p* = 0.0321 vs. control, respectively). However, only the treatment with 250 mg/L of HE was significantly different from the Pb group (*p* = 0.0011) (Figure 4B).

### 2.4. Cyclooxygenases Expression and Activity

#### 2.4.1. *COX-1* mRNA Expression

**Figure 5:**
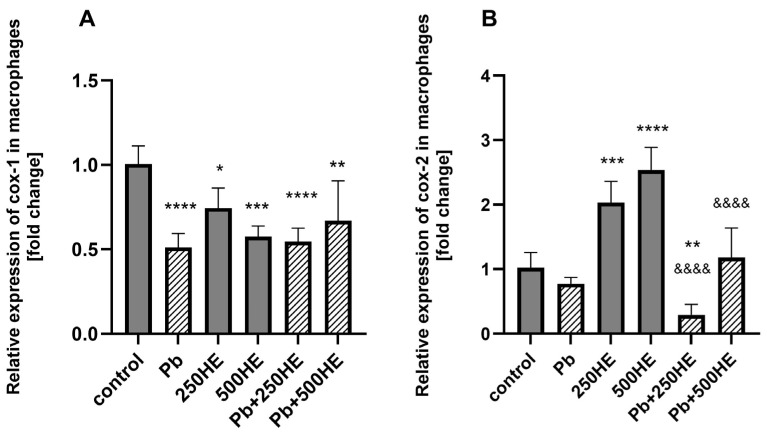
Relative mRNA expression of *COX-1* (**A**) and *COX-2* (**B**) in macrophages of the control group and experimental groups: exposed to 3.5 µg/dL Pb, treated with 250 mg/L or 500 mg/L *Hericium erinaceus* extract (250HE and 500HE), and exposed to 3.5 µg/dL Pb combined with 250 mg/L or 500 mg/L *Hericium erinaceus* extract. Each group included six samples. Data are presented as mean ± SD. One-way ANOVA followed by Tukey’s multiple comparisons post hoc test: * *p* < 0.05, ** *p* < 0.005, *** *p* < 0.0005, **** *p* < 0.0001 vs. control; &&&& *p* < 0.0001 vs. corresponding HE group.

Incubation of macrophages with HE extracts at both 250 mg/L and 500 mg/L significantly decreased *COX-1* mRNA expression by 26% (*p* = 0.0471) and 43% (*p* = 0.0001), respectively. A similar effect was observed in the Pb-treated group (*p* < 0.0001). Co-incubation of macrophages with Pb and HE (250 mg/L or 500 mg/L) also resulted in downregulation of *COX-1* expression (*p* < 0.0001, *p* = 0.0017) (Figure 5A).

#### 2.4.2. *COX-2* mRNA Expression

The addition of HE extract at both 250 mg/L and 500 mg/L resulted in a 99% (*p* = 0.0020) and 148% (*p* < 0.0001) upregulation of *COX-2* mRNA expression, respectively. No significant changes in *COX-2* expression were observed in the Pb-treated group. However, combined treatment with Pb and 250 mg/L HE led to downregulation of *COX-2* expression compared with the control and 250HE group (*p* = 0.0230, *p* < 0.0001, respectively). Moreover, *COX-2* mRNA expression in the Pb + 500HE group was significantly lower compared with both the 500HE group (*p* < 0.0001) and the Pb + 250HE group (*p* = 0.0002) (Figure 5B).

#### 2.4.3. COX-1 Protein Expression Measured by ICC Analysis

**Figure 6:**
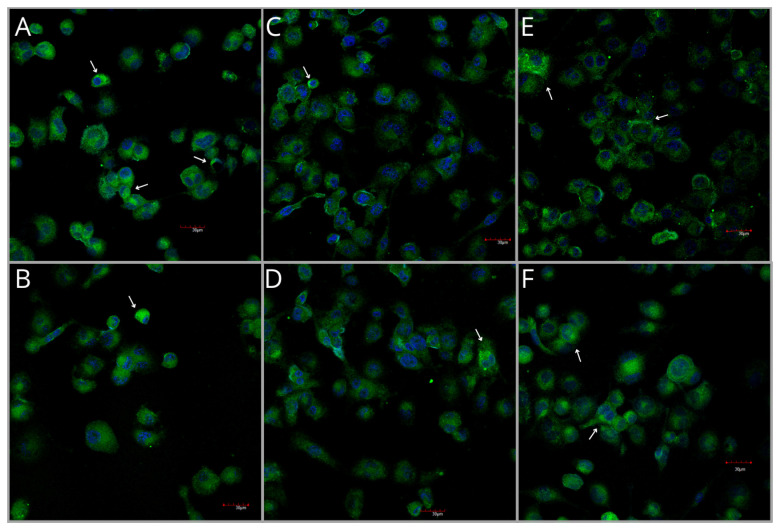
Representative immunocytochemical images showing COX-1 protein expression in THP-1 macrophages from the control group (**A**) and experimental groups: exposed to 3.5 µg/dL Pb (**B**), treated with 250 mg/L (**C**) or 500 mg/L (**D**) *Hericium erinaceus* extract, and exposed to 3.5 µg/dL Pb combined with 250 mg/L (**E**) or 500 mg/L (**F**) *Hericium erinaceus* extract. Each group included six samples. White arrows indicate regions of pronounced immunoreactivity. Scale bar = 30 µm.

**Figure 7:**
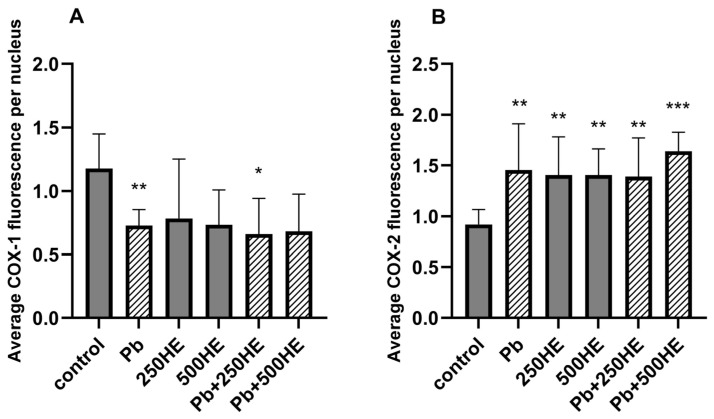
Relative protein expression of COX-1 (**A**) and COX-2 (**B**) in macrophages of the control group and experimental groups: exposed to 3.5 µg/dL Pb, treated with 250 mg/L or 500 mg/L *Hericium erinaceus* extract (250HE and 500HE), and exposed to 3.5 µg/dL Pb combined with 250 mg/L or 500 mg/L *Hericium erinaceus* extract. Each group included six samples. Data are presented as mean ± SD. U Mann–Whitney test: * *p* < 0.05, ** *p* < 0.005, *** *p* < 0.0005 vs. control.

Analysis of COX-1 protein expression in fixed cultures of THP-1 macrophages revealed the highest expression of this enzyme in the control group (Figure 6 and Figure 7A). Immunoexpression of COX-1 was significantly reduced compared with the control in the Pb-exposed group (*p* = 0.0028) and in the Pb-exposed group treated with 250 mg/L of HE (*p* = 0.0204). Other groups also showed a decrease in COX-1 expression compared with the control, although these changes were not statistically significant. Groups treated with both Pb and HE did not differ significantly from those treated with Pb or HE alone (Figure 6 and Figure 7A).

#### 2.4.4. COX-2 Protein Expression Measured by Immunocytochemical (ICC) Analysis

**Figure 8:**
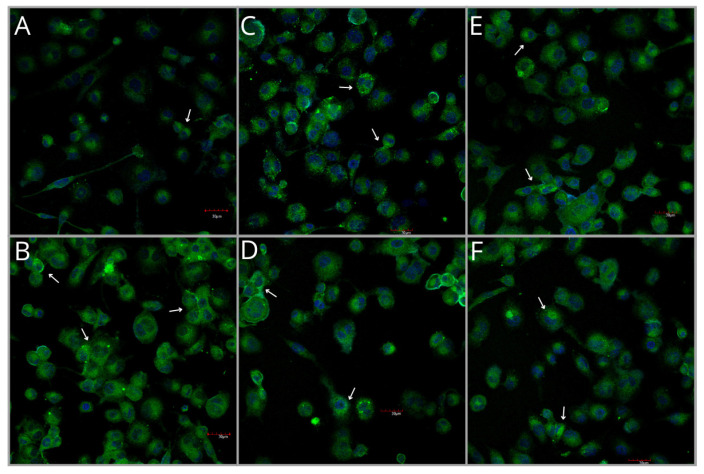
Representative immunocytochemical images showing COX-2 protein expression in THP-1 macrophages from the control group (**A**) and experimental groups: exposed to 3.5 µg/dL Pb (**B**), treated with 250 mg/L (**C**) or 500 mg/L (**D**) *Hericium erinaceus* extract, and exposed to 3.5 µg/dL Pb combined with 250 mg/L (**E**) or 500 mg/L (**F**) *Hericium erinaceus* extract. Each group included six samples. White arrows indicate regions of pronounced immunoreactivity. Scale bar = 30 µm.

Analysis of COX-2 protein expression in fixed cultures of THP-1 macrophages revealed the lowest expression of this enzyme in the control group (Figure 7B and Figure 8). Pb exposure resulted in a 1.4-fold increase in COX-2 expression compared with the control (*p* = 0.0016). Treatment with 250HE increased expression by 33% (*p* = 0.0055), and a similar effect was observed for 500HE (*p* = 0.0054). Groups exposed to both Pb and HE extract also showed higher COX-2 immunoexpression compared with the control (*p* = 0.0078 and *p* < 0.0001, respectively); however, these values did not differ significantly from those observed in groups exposed to Pb alone or treated solely with HE (Figure 7B and Figure 8).

#### 2.4.5. TXB2 Concentration in Macrophage Culture Media

**Figure 9:**
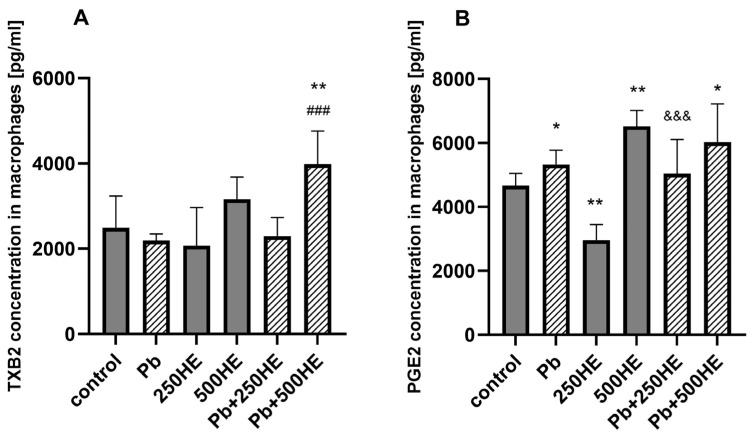
The concentration of TXB2 (**A**) and PGE_2_ (**B**) in macrophage culture medium of the control group and experimental groups: exposed to 3.5 µg/dL Pb, treated with 250 mg/L or 500 mg/L *Hericium erinaceus* extract (250HE and 500HE), and exposed to 3.5 µg/dL Pb combined with 250 mg/L or 500 mg/L *Hericium erinaceus* extract. Each group included six samples. Data are presented as mean ± SD. One-way ANOVA followed by Tukey’s multiple comparisons post hoc test: * *p* < 0.05, ** *p* < 0.005 vs. control; ### *p* < 0.0005 vs. Pb; &&& *p* < 0.0005 vs. corresponding HE group.

Treatment with Pb or HE alone did not alter the concentration of TXB_2_ in the culture medium of THP-1 macrophages. An increase in TXB_2_ production was observed only in the group exposed to Pb and treated with a higher concentration of HE extract (500 mg/L), compared with the control (*p* = 0.0042) and Pb-only groups (*p* = 0.0004) (Figure 9A).

#### 2.4.6. PGE_2_ Concentration in Macrophage Culture Media

Pb treatment (3.5 µg/dL) induced an increase in PGE_2_ synthesis by THP-1 macrophages (*p* = 0.0480 vs. control). Treatment with 250 mg/L HE reduced the level of this mediator (*p* = 0.0062), whereas a higher concentration (500 mg/L) led to increased PGE_2_ synthesis (*p* = 0.0023 vs. control). In Pb-exposed groups, PGE_2_ levels were different from the control group only in the group treated with 500 mg/L HE (*p* = 0.0415); however, no changes were observed vs. the Pb-only group. PGE_2_ concentrations were higher in the Pb + 250HE group compared with the corresponding group not exposed to Pb (*p* = 0.0006) (Figure 9B).

## 3. Discussion

*Hericium erinaceus* is a medicinal mushroom with proven neurotropic properties [33]. In our study, HE exhibited immunomodulatory and neuromodulatory potential through the modulation of prostaglandin synthesis and pro-inflammatory mediators in a microglia-oriented in vitro model of THP-1 macrophages under Pb-induced stress conditions.

### 3.1. The Modulation of Neuroinflammatory Signaling by Hericium Erinacues

Within our experimental model, exposure to HE was associated with elevated TNF alpha levels. Beyond its pro-inflammatory properties, TNF alpha may also exert beneficial effects within the central nervous system. By activating tumor necrosis factor receptor 2 (TNFR2) on neurons and astrocytes, TNF alpha promotes neurogenesis and neuroprotection [39], and induces the expression of pro-survival factors, including brain-derived neurotrophic factor (BDNF), thereby supporting synaptic network repair in damaged brain regions [40,41,42,43]. Therefore, in our model, the observed increase in TNF alpha induced by the HE extract may exert beneficial effects on central nervous system function. Moreover, TNF alpha may modulate neuronal autophagy via NF-κB and mechanistic target of rapamycin (mTOR) signaling, facilitating the removal of damaged organelles and proteins and preventing the accumulation of toxic protein aggregates characteristic of neurodegenerative diseases [44]. In addition, low levels of TNF alpha (up to 10 ng/mL) mobilized microglia and astrocytes to jointly release growth factors and antioxidants, thereby maintaining a balance between inflammatory activity and reparative processes [39,45,46,47]. Similarly, in our experiment, TNF alpha concentrations in the HE-treated groups remained below 10 ng/mL, reaching a maximum of 7.4 mg/L in the 250HE group and 5.6 mg/L in the 500HE group. These levels are consistent with a regulatory, rather than overtly pro-inflammatory, role of TNF alpha in the experimental model used. Importantly, the biological effects of TNF alpha were highly context-dependent and may shift from neuroprotective to neurotoxic depending on concentration, duration of exposure, and receptor-specific signaling.

In the present study, in HE-treated cells, *COX-1* gene expression was downregulated in favor of *COX-2* expression, which was also evident at the protein level, as well as in increased PGE_2_ secretion. Under stress conditions, COX-2 primarily contributes to the synthesis of pro-inflammatory mediators, compensating for constitutive COX-1 activity [48]. Such a shift is characteristic of an adaptive immune response, enabling macrophages to respond dynamically to environmental and inflammatory stimuli. In the presence of elevated TNF alpha levels, HE-induced COX-2 upregulation may occur via an NF-κB-dependent pathway [49,50].

Previous studies have shown that cell cultures exposed to HE extracts exhibit decreased COX-2 and TNF alpha levels, inhibited TNF alpha-induced angiogenesis and ROS generation in human EA.hy926 endothelial cells [34], as well as reduced COX-2 expression in astrocytes of C57BL mice [35]. In contrast, in our model, exposure to *Hericium erinaceus* extract resulted in increased COX-2 expression in THP-1 macrophages. This difference may be due to variations in cellular context, as previous studies examined endothelial cells or astrocytes, whereas the present model involves microglia-like cells exposed to a Pb-modified environment, as well as differences in dosing. To date, the effects of this extract on macrophages have not been investigated.

Moreover, in our experiments, treatment with the 500 mg/L HE extract resulted in increased PGE_2_ levels up to a mean concentration of 6524.2 pg/mL. Microglia are specialized non-neuronal cells whose primary role is to maintain CNS homeostasis. Due to their unique potassium channels, these cells are highly sensitive to even minor pathological changes within the nervous system [51]. Consequently, exposure to a novel stimulus such as HE extract can trigger an immunomodulatory response in microglia, a response that was also observed in our experiments using THP-1 macrophages. In our study, this effect was dose-dependent, with increasing HE concentrations resulting in stronger induction of COX-2 expression and activity. Nevertheless, we observed elongated morphology, reduced cytoplasmic granularity, and more organized cellular architecture, which suggest that treatment with HE extract may promote macrophage polarization toward a less pro-inflammatory phenotype [52,53], although polarization markers were not directly evaluated in this study.

Although PGE_2_ and COX-2 are commonly associated with pro-inflammatory signaling, it also exerts important regulatory functions in the CNS by modulating the magnitude and resolution of microglial responses [54,55]. PGE_2_ has been shown to reduce microglia-mediated neuronal toxicity through interactions with E-prostanoid (EP) receptors EP2 and EP4 [55,56]. This interaction leads to decreased nitric oxide production [53] and reduced IL-1β release [55,57]. By acting on microglia and other CNS cells, PGE_2_ modulates cellular activity and prevents excessive inflammatory responses, thereby contributing to the protection of neural tissue [56,57]. Moreover, PGE_2_ suppresses both the transcription and translation (mostly) of inflammatory mediators such as TNF alpha, IL-6, and macrophage inflammatory protein 1 alpha (MIP-1α) [57,58], and therefore might modulate the activation of key signaling pathways, including NF-κB and MAPK, in microglial cells [59].

Moreover, previous studies have demonstrated that compounds derived from *H. erinaceus*, including erinacine C, hericerin, isohericerinol A, and corallocin A, upregulate BDNF expression. Among these, isohericerinol A and corallocin A induce particularly pronounced increases in BDNF levels [33]. BDNF, through binding to tropomyosin receptor kinase B (TrkB) receptors, activates the rat sarcoma proto-oncogene (Ras)/rapidly accelerated fibrosarcoma kinase (RAF)/mitogen-activated protein kinase (MAPK), phospholipase C gamma (PLCγ), and phosphoinositide 3-kinase (PI3K)/protein kinase B (Akt) signaling cascades, which are critical for neurogenesis, neuronal survival, and synaptic plasticity [60,61,62]. Reduced BDNF expression has been demonstrated in patients with Alzheimer’s disease, particularly in the hippocampus, dentate gyrus, neocortex, and the nucleus basalis of Meynert [33,63].

BDNF secretion is, in turn, regulated by COX-2 expression. PGE_2_ generated via COX-2 signaling acts through EP2 receptors, activating the cyclic adenosine monophosphate/protein kinase A (cAMP/PKA) pathway and increasing BDNF levels in the hippocampus of mice and rats following prolonged seizures [64]. Although BDNF levels were not directly measured in the present study, the observed activation of the COX-2/PGE_2_ axis suggests a potential mechanistic link to neurotrophic signaling reported in earlier experimental models.

PGE_2_ is therefore involved in regulating inflammatory processes and intercellular communication within the central nervous system. Through the synthesis and release of PGE_2_, microglia can modulate the activity of neurons and astrocytes, thereby influencing their contribution to the maintenance of neuronal homeostasis [55,56]. Accordingly, PGE_2_ released by microglia, as observed in our THP-1-derived macrophage model, might be important in coordinating inflammatory responses, and given the neuroprotective properties of BDNF, it might affect the functions of both glial and other cell types in the brain.

### 3.2. Lead-Induced Immune Dysregulation

Our results showed that exposure to low levels of Pb (3.5 µg/dL) for 48 h did not alter TNF alpha concentration or its gene expression. Pb exhibits inhibitory properties that impair enzymatic activity and disrupt the binding of transcriptional regulatory proteins by targeting their zinc finger domains [65,66]. At the same time, this element is a well-known factor that potentiates inflammatory processes, including neuroinflammation [3]. However, isolated exposure to this element, particularly at low levels, is often insufficient to induce an increase in the concentration of this cytokine, whereas co-exposure with additional stimuli is required [67], as also observed in our study. Thus, at low concentrations, Pb appears to act primarily as an immune-disrupting factor that alters the quality and coordination of inflammatory signaling rather than directly amplifying cytokine production. Reports in the literature are consistent with our findings. Under conditions of low and isolated exposure in our experiment, Pb did not directly elicit a cytokine response (IL-6, TNF alpha) but instead acted as an immunomodulatory factor, with its effects becoming apparent only in the presence of additional stimuli.

In the literature, TNF alpha expression has been reported to be variably affected by Pb exposure [68]. Notably, in individuals exposed to Pb with high average Pb concentrations, a significant decrease in circulating TNF alpha levels has been observed, suggesting that long-term Pb exposure may induce adaptive mechanisms that downregulate basal TNF alpha release [66,68,69]. Nevertheless, despite the absence of TNF alpha upregulation, our morphological observations indicate that macrophages exposed even to low levels of Pb tend to polarize toward an inflammatory-like phenotype.

Additionally, Pb exposure resulted in decreased *COX-1* gene and protein expression, while simultaneously increasing the expression of the inducible COX-2 enzyme and causing a slight increase in its product, PGE_2_. However, after 48 h, no increase in *COX-2* gene expression was observed. The absence of a concomitant increase in TNF alpha may suggest that COX-2 upregulation in the presence of low Pb concentrations does not occur via an NF-κB-dependent pathway, but is instead regulated at the translational level [70]. Wei et al. demonstrated that, in glial cells, induction of the cyclooxygenase pathway is modulated by nuclear factor of activated T cells (NFAT) transcription factors rather than by the AP-1/NF-κB pathway [71]. It is also possible that in our experiment, changes at the gene expression level occurred earlier during the experiment and had already subsided by the 48-h time point.

### 3.3. Immunomodulatory Action of Hericium erinaceus in THP-1 Macrophages Under Low-Level Lead Exposure

Our further evaluation of the macrophage response to combined exposure to Pb and HE extract demonstrated that, under conditions of low Pb exposure (3.5 µg/dL), HE at the concentrations used in our study (250 mg/L and 500 mg/L) did not upregulate *COX-2* gene expression and, in fact, reduced it in the group treated with the lower HE dose. COX-2 protein expression was elevated in both groups; however, a significant increase in PGE_2_ synthesis was observed only in the group treated with the higher HE concentration. Notably, treatment with a lower concentration of HE in the presence of Pb modulated the secretion of this mediator, restoring its levels to those observed in untreated control cells. These findings indicate a strongly dose-dependent effect of HE.

At the same time, we have observed increased synthesis and expression of TNF alpha in 500HE vs. control and Pb groups; however, TNF alpha levels remained lower than those detected in the corresponding HE-only groups. Similar results were observed in gene expression level, particularly in the group incubated with the lower HE dose.

In addition to TNF alpha, in our study, the treatment with *Hericium erinaceus* of Pb-exposed cells resulted in increased IL-6 levels. IL-6 represents a pleiotropic cytokine that links inflammatory activation with neurotrophic and regenerative processes; depending on the signaling context, IL-6 may support neuronal survival and glial communication rather than drive chronic neuroinflammation [72]. Our observation suggests that low-level Pb exposure may prime THP-1 cells, thereby unmasking the immunomodulatory action of *Hericium erinaceus* that is not evident under basal conditions.

Furthermore, exposure to Pb alone reduced MCP-1 levels, whereas treatment with HE increased MCP-1 secretion; notably, under combined Pb and HE exposure, MCP-1 levels were restored toward control values, suggesting a normalization of chemokine signaling rather than nonspecific pro-inflammatory activation. Consistent with these findings, our morphological observations indicate that HE extract mitigated Pb-induced macrophage polarization, which may suggest a shift toward a less pro-inflammatory phenotype. Beyond its role in leukocyte chemotaxis, MCP-1 contributes to the coordination of inflammatory responses and tissue remodeling [73]. It mediates neuroprotective actions of neurotransmitters, and its expression is essential for appropriate immune surveillance without excessive inflammatory activation [73,74,75]. Thus, normalization of MCP-1 levels under combined Pb and *Hericium erinaceus* exposure may reflect controlled chemokine signaling rather than pro-inflammatory polarization.

## 4. Materials and Methods

### 4.1. Reagents and Equipment Used in the Study

Reagents used in the study were as follows: Roswell Park Memorial Institute (RPMI) 1640 medium (GeneDireX, Taoyuan, Taiwan; cat. CC142-0500); fetal bovine serum (FBS) (Merck, KGaA, Darmstadt, Germany; cat. F7524); penicillin, and streptomycin (GeneDireX; Taoyuan, Taiwan; cat. CC502-0100); culture plates (Starlab International, Hamburg, Germany); phorbol 12-myristate 13-acetate (PMA) (Merck KGaA, Darmstadt, Germany; cat. P1585); GeneMatrix Universal RNA 473 Purification Kit (EURx, Gdańsk, Poland; cat. E3598); DNase I enzyme (EURx, Gdańsk, Poland; cat. E1345); RevertAid RT Kit (ThermoFisher Scientific, Waltham, MA, USA; cat. K1622); SensiFAST™ Sybr^®^ No-ROX kit (Meridian Bioscience, Cincinnati, OH, USA; cat. BIO-98005); AlphaLISA Human TNF alpha Detection Kit (Revvity, Waltham, MA, USA; cat. AL3157HV); PGE_2_ enzyme immunoassay kit (FineTest, Wuhan, China; cat. EH4233); TXB_2_ enzyme immunoassay kit (FineTest, Wuhan, China; cat. EP0162); MCP-1 enzyme immunoassay kit (FineTest, Wuhan, China; cat. EH0222); IL-6 enzyme immunoassay kit (FineTest, Wuhan, China; cat. EH0201); Triton X-100 (Sigma-Aldrich, Poznań, Poland; cat. X100); primary mouse antibody against COX-1 (Abcam, Cambridge, UK; cat. ab81296); primary mouse antibody against COX-2 (Abcam, Cambridge, UK; cat. ab6233); FITC-conjugated anti-rabbit IgG secondary antibody (Sigma-Aldrich, Poznań, Poland; cat. F0382); antibody diluent (Agilent Dako, Santa Clara, CA, USA; cat. S2022); Hoechst 33258 (ThermoFisher Scientific, Waltham, MA, USA; cat. H3569).

Equipment used in the study included: NanoDrop™ 2000 Spectrophotometer (Thermo Fisher Scientific, Waltham, MA, USA); confocal laser scanning microscope, FV1000 system coupled to an IX81 inverted microscope (Olympus, Hamburg, Germany); CFX96 Touch Real-Time PCR Detection System (Bio-Rad, Hercules, CA, USA); Alpha-compatible microplate reader VICTOR Nivo (Revvity, Waltham, MA, USA).

Software used in the study included: Statistica 10 (StatSoft, Kraków, Poland); GraphPad Prism 10.6.1 (GraphPad Software, Boston, MA, USA).

### 4.2. Preparation of Hericium erinaceus Extract

The ethanolic extract of *Hericium erinaceus* was prepared using a modified protocol based on Kushairi et al. [76]. Dried fruiting bodies were finely ground with a mortar and pestle, and the resulting powder was extracted with 80% (*v*/*v*) aqueous ethanol at a solid-to-solvent ratio of 1:10 (*w*/*v*) for 72 h at room temperature. The solvent phase was collected and filtered every 24 h, and the residual material was subjected to two additional extraction cycles under identical conditions. The pooled filtrates were concentrated under reduced pressure using a rotary evaporator to obtain the crude ethanolic extract, which was then stored at −80 °C until further use.

### 4.3. Cell Culture and Treatment

Human macrophages were derived from the THP-1 monocytic cell line. Cells were cultured in RPMI 1640 medium (GeneDireX, Taoyuan, Taiwan; cat. CC112-0500) supplemented with 10% fetal bovine serum (FBS; Merck KGaA, Darmstadt, Germany; cat. F7524), 100 IU/mL penicillin, and 10 µg/mL streptomycin (GeneDireX; Taoyuan, Taiwan, cat. CC502-0100). The cultures were maintained in a humidified incubator at 37 °C in an atmosphere containing 5% CO_2_.

First, HE cytotoxicity was assessed in THP-1 macrophages using the WST-1 assay. Cells were seeded in 96-well plates at an initial density of 0.5 × 10^6^ cells/mL (Starlab International, Hamburg, Germany). Differentiation into macrophage-like cells was induced by treatment with 100 nM PMA (Merck KGaA, Darmstadt, Germany; cat. P1585) for 24 h, followed by replacement with fresh medium and a 24-h resting period. After resting, the medium was replaced with fresh medium containing HE extract at concentrations of 0, 50, 100, 250, 500, 1000, or 2000 mg/L. Cells were then incubated for an additional 24 h, washed, and cytotoxicity was evaluated using the WST-1 reagent (Cell Proliferation Reagent WST-1; Merck KGaA, Darmstadt, Germany; cat. 5015944001) according to the manufacturer’s instructions. Each concentration was tested in five replicates.

For the experiments, THP-1 cells were seeded as described above, differentiated into macrophages using PMA (as described above), and allowed to rest for an additional 24 h.

In the initial experimental setup, THP-1-derived macrophages were cultured for 48 h in complete medium and assigned to the following treatment groups:

(A) Control—cells cultured without any additives (*n* = 6);
(B) Pb—cells treated with 3.5 µg/dL lead acetate;
(C) 250HE—cells treated with 250 mg/L HE extract;
(D) 500HE—cells treated with 500 mg/L HE extract;
(E) Pb + 250HE—cells treated with 3.5 µg/dL lead acetate and 250 mg/L HE extract;
(F) Pb + 500HE—cells treated with 3.5 µg/dL lead acetate and 500 mg/L HE extract.

HE concentrations were selected based on our cytotoxicity assays and previous reports in the literature [75].

After incubation, the cells were gently harvested by scraping, and the suspensions were centrifuged at 800× *g* for 10 min to obtain cell pellets. Both the collected cell pellets and culture medium samples were subsequently stored at −80 °C until further analyses.

### 4.4. Gene Expression Analysis of TNF alpha, COX-1, and COX-2 Genes Using Rt-Pcr

#### 4.4.1. Total RNA Isolation

Total RNA was isolated from the cell cultures using the GeneMatrix Universal RNA Purification Kit (EURx, Gdańsk, Poland, cat. E3598) according to the manufacturer’s protocol. RNA samples were purified of any remaining DNA by treatment with DNase I enzyme (EURx, Gdańsk, Poland, cat. E1345). The RNA concentration and purity of each sample were evaluated using a NanoDrop™ 2000 Spectrophotometer (Thermo Fisher Scientific, Waltham, MA, USA).

#### 4.4.2. Gene Expression Determination

First-strand cDNA of each sample was synthesized from 2 µg of DNase-treated total RNA in a 20 µL reaction volume, using the RevertAid RT Kit (ThermoFisher Scientific, Waltham, MA, USA, cat. K1622) according to the manufacturer’s protocol. cDNA samples were diluted 10× with nuclease-free water and stored at −20 °C for further analysis.

Amplification of selected genes from a cDNA template was performed by qPCR using the SensiFAST™ Sybr^®^ No-ROX kit (Meridian Bioscience, Cincinnati, OH, USA, cat. BIO-98005) on a CFX96 Touch Real-Time PCR Detection System (Bio-Rad, Hercules, CA, USA). The primers used in the qPCR analyses are listed in Table 1. They were designed using Primer-Blast. To eliminate genomic DNA amplification, the primers were designed to span an exon-exon junction of the analyzed genes. Cycling conditions (temperature and time) were determined according to the manufacturer’s instructions, considering the melting temperatures of the primers and the length of the expected amplicons. Additionally, to exclude nonspecific products, the melting curves of the PCR products were analyzed after terminating the reaction. The reaction products obtained using each pair of primers were sequenced to confirm the results. The amplification reactions were verified to ensure that the efficiency was within the range of 95–105% for all genes tested. Therefore, Livak’s comparative method (∆∆Ct) was used to calculate the fold change in gene expression normalized to the average of two reference genes: *GAPDH* and *RACK1*. 

### 4.5. TNF Alpha Concentration Determination Using AlphaLISA

The concentration of TNF alpha in the cell culture supernatants was determined using the AlphaLISA Human TNF alpha Detection Kit (Revvity, Waltham, MA, USA; cat. AL3157HV), following the manufacturer’s protocol. Briefly, samples and TNF alpha standards were incubated with a biotinylated anti-TNF alpha antibody together with donor and acceptor beads coated with TNF alpha-specific antibodies. During the incubation, the formation of immunocomplexes induced a proximity-based luminescent signal, which was measured at 615 nm using an Alpha-compatible microplate reader VICTOR Nivo (Revvity, Waltham, MA, USA). The emitted signal intensity was directly proportional to the concentration of TNF alpha in the tested samples.

### 4.6. MCP-1, IL-6, PGE_2_ and TXB2 Concentration Determination Using Elisa

The activity of cyclooxygenases COX-1 and COX-2 was assessed indirectly by measuring their respective metabolites—prostaglandin E_2_ (PGE_2_) and thromboxane B_2_ (TXB_2_). The concentrations of PGE_2_ (FineTest, Wuhan, China; cat. EH4233) and TXB_2_ (FineTest, Wuhan, China; cat. EP0162) in the culture media were determined spectrophotometrically using enzyme immunoassay kits, following the manufacturer’s instructions. In the same manner, the concentrations of the chemokine MCP-1 (FineTest, Wuhan, China; cat. EH0222) and the cytokine IL-6 (FineTest, Wuhan, China; cat. EH0201) in the culture media were determined.

### 4.7. Imaging of Cyclooxygenase-1 and Cyclooxygenase-2 Expression

Cells were seeded onto microscope slides as described in Section 4.3, Cell culture and treatment, using 6-well plates. After cultivation, they were gently rinsed with PBS and fixed in 4% buffered formaldehyde for 10 min at room temperature. Following fixation and washing with PBS, cell membranes were permeabilized using 0.5% Triton X-100 (Sigma-Aldrich, Poznań, Poland; cat. X100) in PBS. The samples were then washed again and incubated overnight at 4 °C with primary mouse antibodies against COX-1 and COX-2 (Abcam, Cambridge, UK; cat. ab81296 and ab62331) diluted 1:60. Subsequently, the cells were washed and exposed for 30 min at room temperature to a FITC-conjugated anti-rabbit IgG secondary antibody (Sigma-Aldrich, Poznań, Poland; cat. F0382) diluted 1:60 in antibody diluent (Agilent Dako, Santa Clara, CA, USA, cat. S2022). After final PBS rinses, nuclear staining was performed using Hoechst 33258 for 30 min at room temperature.

Fluorescence analysis was conducted with a confocal laser scanning microscope (FV1000 system coupled to an IX81 inverted microscope, Olympus, Hamburg, Germany). Sequential scanning in three channels was employed to optimize the detection of Hoechst 33258 and FITC signals. Fluorescent and transmitted light images were merged for enhanced visualization, and fluorescence intensity was quantified using ImageJ 1.54g software [77].

### 4.8. Statistical Analysis

Statistical analyses were carried out using Statistica 10 software (StatSoft, Poland) and GraphPad Prism (GraphPad Software, Boston, MA, USA). Data are presented as the arithmetic mean ± standard deviation (SD). The normality of data distribution was evaluated using the Shapiro–Wilk W test. Comparisons between multiple groups were performed using one-way ANOVA followed by Tukey’s multiple comparisons post hoc test for normally distributed data, while the Mann–Whitney U test was applied for nonparametric data. A *p*-value < 0.05 was considered statistically significant.

## 5. Conclusions

Our findings indicate that the HE extract does not promote inflammatory processes but instead acts as a regulator of inflammatory signaling. This effect is mediated, at least in part, by the restoration of appropriate macrophage responsiveness, which supports repair mechanisms while preventing excessive cellular activation. In Pb-exposed THP-1 cells, HE appears to modulate the COX-2/PGE_2_ pathway and cytokine production in the presented THP-1 macrophage model, potentially limiting the development of a pro-inflammatory macrophage phenotype. The reduced synthesis of PGE_2_ and cytokines such as IL-6 and TNF alpha may further contribute to the maintenance of homeostasis not only in microglia but also in other central nervous system cells, including astrocytes and neurons. In addition, HE treatment may mitigate the immunomodulatory effects of low-level Pb exposure. By preserving microglial immunomodulatory capacity, HE may also support BDNF-regulated neuroprotective signaling pathways. Although Pb exposure is known to induce neurodegenerative changes, our results suggest that *Hericium erinaceus* exerts protective effects by restoring appropriate macrophage activity and modulating immune processes in a microglia-like model of low-level Pb-exposed THP-1 macrophages.

### Limitations of the Study

This experiment was conducted at a single time point, which limited insight into early gene expression changes following stimulus exposure, especially for genes that respond quickly, such as *COX-2* and *TNF alpha*. Analysis across several time points would allow a more thorough evaluation of our hypotheses regarding the dynamics of gene expression. In addition, our analyses were restricted to a single low Pb concentration, whereas Pb exposure in the general population occurs across a broad range of levels. Evaluation of higher concentrations would help determine whether the observed effects are consistent across different exposure intensities.

The HE concentrations applied in this study were selected based on our cytotoxicity analyses and previous reports; however, given the dose-dependent effects observed, alternative concentrations of *Hericium erinaceus* may modulate the inflammatory response differently. For the study, we chose the doses well tolerated in vitro; however, additional research is needed to strengthen the translational relevance of our observations.

Moreover, the morphological analysis performed was qualitative rather than quantitative. We have analyzed the effects of the whole extract, not individual compounds, and mushroom extracts may contain endotoxin contaminants, which can interfere with inflammatory responses. Consequently, further studies should include quantitative assessment of macrophage polarization markers, together with a broader panel of markers associated with intermediary signaling pathways, to substantiate the immunomodulatory properties of the HE.

Although the model employed is widely used as a microglia-like system, it does not fully recapitulate the biology of resident microglia, which are the native immune cells of the central nervous system. These cell types arise from distinct progenitors, and THP-1 cells lack specific glial markers and features. Nevertheless, they share similarities in inflammatory signaling. Finally, residual PMA activation may have influenced baseline cytokine levels; however, all experimental groups were exposed to PMA under the same conditions.

## Figures and Tables

**Table 1:** qPCR primers used in this study.

Gene	Forward Primer	Reverse Primer	Amplicon Length (bp)	T_M_ of the Amplification Products (°C) ^1^
*TNF alpha*	AGCCCATGTTGTAGCAAACCC	GGACCTGGGAGTAGATGAGGT	149	87
*COX-1*	TTGGGCCATGGGGTAGACCT	CGAGGGCGGGTACATTTCTC	126	83.5
*COX-2*	CCCTTCTGCCTGACACCTTT	TTCTGTACTGCGGGTGGAAC	172	81.5
*GAPDH*	ATGCCTCCTGCACCACCAACT	ATGGCATGGACTGTGGTCATGAGT	97	83.5
*RACK1*	GAGTGTGGCCTTCTCCTCTG	GCTTGCAGTTAGCCAGGTTC	224	84.5

^1^ TM melting temperature.

## Data Availability

All data generated during this study are included in this published article. Additional raw data are available from the authors upon reasonable request.

## Abbreviations

The following abbreviations are used in this manuscript:

Aβ	beta-amyloid
Akt	protein kinase B
BDNF	brain-derived neurotrophic factor
cAMP/PKA	cyclic adenosine monophosphate/protein kinase A
CDC	Centers for Disease Control and Prevention
CNS	central nervous system
COX	Cyclooxygenases
COX-1	cyclooxygenase-1
COX-2	cyclooxygenase-2
EP	E-prostanoid receptors
HE	*Hericium erinaceus*
IL	interleukins
MAPK	mitogen-activated protein kinase
MCP-1	monocyte chemoattractant protein-1
MIP-1α	macrophage inflammatory protein 1 alpha
MMP-9	matrix metalloproteinase-9
mTOR	mechanistic target of rapamycin
NF-κB	nuclear factor kappa-B
NFAT	nuclear factor of activated T cells
NGF	nerve growth factor
Nrf2	nuclear factor erythroid 2-related factor 2
Pb	lead
PGE_2_	prostaglandin E_2_
PI3K	phosphoinositide 3-kinase
PLCγ	phospholipase C gamma
PMA	phorbol 12-myristate 13-acetate
RAF	rapidly accelerated fibrosarcoma kinase
Ras	rat sarcoma proto-oncogene
ROS	reactive oxygen species
RPMI	Roswell Park Memorial Institute
TGFβ	transforming growth factor beta
TNF alpha	tumor necrosis factor alpha
TNFR2	tumor necrosis factor receptor 2
TrkB	tropomyosin receptor kinase B
TXA_2_	thromboxane A_2_
TXB_2_	thromboxane B_2_
